# Perceptions and awareness of endocrine disruptors among mothers in Serbia and health implications

**DOI:** 10.1530/EC-24-0678

**Published:** 2025-02-14

**Authors:** Katarina Živančević, Đurđica Marić, Luka Manić, Vera Bonderović, Jovana Živanović, Danijela Djukic-Cosic, Zorica Bulat, Biljana Antonijevic, Zoran Vilendecic, Sandra Ilić, Iris Žeželj, Aleksandra Buha Djordjevic

**Affiliations:** ^1^ Department of Toxicology “Akademik Danilo Soldatović”, University of Belgrade – Faculty of Pharmacy, Belgrade, Serbia; ^2^ University of Belgrade – Faculty of Biology, Institute of Physiology and Biochemistry “Ivan Djaja”, Department of General Physiology and Biophysics, Center for Laser Microscopy, Belgrade, Serbia; ^3^ Clinic for Gynecology and Obstretics, University Clinical Center of Serbia, Belgrade, Serbia; ^4^ School of Medicine, University of Belgrade, Belgrade, Serbia; ^5^ Psychology Department, Faculty of Philosophy, University of Belgrade, Belgrade, Serbia; ^6^ Laboratory for Experimental Psychology, Faculty of Philosophy, University of Belgrade, Belgrade, Serbia; ^7^ LIRA laboratory, Faculty of Philosophy, University of Belgrade, Belgrade, Serbia

**Keywords:** endocrine disruptors, awareness, survey, health, mothers

## Abstract

Endocrine-disrupting chemicals are significant contributors to various detrimental conditions, mechanistically disrupting the endocrine system and causing adverse health effects. Mounting evidence suggests they can induce multigenerational and transgenerational effects, yet awareness among individuals remain insufficient. This study aimed to assess the knowledge, attitudes and ways of informing mothers in Serbia about endocrine disruptors based on information from 190 women in Serbia. The research was conducted using a survey consisting of multiple-choice questions comprising: the first part aimed to collect sociodemographic data, the second part related to knowledge and attitudes about endocrine disruptors, and the third part focused on the sources of information about endocrine disruptors. Cronbach’s alpha was used to check for scale reliability, and Pearson correlation was used to test the relations between interval variables. ANOVA was employed to test for group differences. The results indicated that mothers in Serbia do not have adequate knowledge about endocrine disruptors (potential sources, categories of substances and alternatives) nor confidence in their ability to mitigate exposure to endocrine disruptors. Also, the estimation of the health risks of exposure to endocrine disruptors was perceived as high, and the mothers thought that they should get additional information about endocrine disruptors before pregnancy. Although with several limitations (i.e. mothers were recruited among those with higher education and mainly from urban areas), the study results highlight the necessity for enhanced maternal education in Serbia regarding endocrine disruptors. Health professionals are deemed most suitable for providing this education, given the respondents' high level of trust in them.

## Introduction

The effects of environmental pollution on human health are receiving increased attention worldwide due to the increased development of non-communicable diseases (1). Among many others, endocrine-disrupting chemicals (EDCs) are recognized as significant contributors to the incidence/occurrence of various detrimental conditions (2). Endocrine disruptors can have a variety of origins, compositions and purposes. They can perturb the endocrine system by imitating the actions of naturally occurring hormones, obstructing the effects of internally produced hormones, altering the synthesis and functionality of hormone receptors, and affecting the production, transportation, metabolism and release of hormones. There is mounting evidence to suggest that endocrine disruptors can produce both multigenerational and transgenerational effects through a sequence of epigenetic alterations (3, 4, 5, 6).

There are over 1400 chemicals with evidence of potential endocrine-disrupting properties (7). They are omnipresent in everyday consumer products. They can be detected in plastic items, including children’s toys and food packaging (including bisphenols, phthalates and phenols), as well as in electronic devices, furniture and construction materials (involving organobromine flame retardants and polychlorinated biphenyls (PCBs)). Furthermore, they are also found in cosmetics, household cleaning agents and personal care items (such as parabens, phthalates, bisphenols, perfluoroalkyl and polyfluoroalkyl substances (PFAS) and toxic metals). In addition, specific pesticides utilized in agriculture, like chlorpyrifos, possess endocrine-disruptive properties (8, 9, 10). Moreover, drugs of the ‘synthetic hormones’ type, such as diethylstilbestrol and oral contraceptives, have also been linked to a range of adverse health outcomes in offspring (5, 6, 11, 12).

Certain endocrine disruptors can accumulate in the environment and the human body, posing health risks (13, 14). Although the body encounters these chemicals regularly at low doses, the effects remain unclear (8). Exposure occurs through food, water, air and skin absorption, leading to prolonged presence and accumulation (9). Combined exposure to multiple chemicals can cause a stronger harmful effect than individual substances (8). Endocrine disruptors are linked to obesity, diabetes, cancers, thyroid dysfunction and neurological issues (4, 9, 15). However, the primary adverse effects caused by endocrine disruptors predominantly refer to male and female reproductive health (16, 17), including menstrual cycle disorders, endometriosis, fertility issues and testicular dysgenesis syndrome (3, 9, 18, 19). The harmful effects of endocrine disruptors on the organism largely depend on the age of exposure, and the most severe adverse consequences will initially appear in the most susceptible populations (13). In this context, pregnancy represents a distinctive period of highly synchronized hormone-regulated processes that substantially change maternal physiology and lead to a particular sensitivity of both mother and fetus (20). Due to their persistent effects, endocrine disruptors have the potential to impact fetal development not only in late pregnancy but also during the early stages, with the adverse consequences of prenatal exposure to endocrine disruptors becoming evident years later (13).

Previous studies reveal a gap between experts' understanding of scientific results and the public’s ability to interpret them. Awareness and knowledge about endocrine disruptors are often lacking, mainly due to insufficient information about the chemicals in everyday products (21). In addition, this lack of awareness stems from knowledge gaps of the potentially harmful consequences that exposure to endocrine disruptors can lead to (9). Timely and precise information concerning endocrine disruptors is essential for specific demographic groups, among which pregnant women hold a significant position due to their impact on the health and well-being of their offspring (22). Consequently, disseminating knowledge and information within this cohort assumes paramount significance.

This study aimed to assess the knowledge, attitudes and ways of informing mothers in Serbia about endocrine disruptors.

## Materials and methods

### Study design and participants

We conducted a cross-sectional study by surveying participants enrolled in the study through convenience sampling. We only enrolled women residing in Serbia aged 18 or older at the time of enrollment who consented to participate in the study. Only women capable of consenting and completing the survey on their own were enrolled. We imposed no additional inclusion or exclusion criteria.

Within the framework of cooperation with the Center for Moms, mothers and future mothers were surveyed. The survey was voluntary and anonymous, distributed to respondents via established online channels in June–September 2023. Respondents were not identified, but we allowed for only one set of submitted answers per respondent. Recorded responses were checked, and no duplicate responses were identified.

Before conducting the research, ethical permission was obtained from the Ethics Committee for Biomedical Research of the Faculty of Pharmacy (no 220/2).

### Sample

The sample comprised adult women from Serbia, aged 22–59 (*n* = 190, Mage = 38.34 and SDage = 6.309). Convenience sampling was used to recruit women who participated in the research. Only women residing in Serbia aged 18 or older were enrolled, and there were no other restrictions to any variable. Most of the participants spent most of their lives in an urban area (87.4%), while the remainder of the sample spent most of their lives in a rural area (12.6%). The sample structure by the highest level of education completed is presented in Table 1.

**Table 1:** Sample structure according to the education level.

Highest completed level of education	% of the sample
Primary school	0
High school	15.8
Student	4.7
College	14.7
Undergraduate University studies or Master studies	58.9
Doctoral studies	5.8

### Coding and analyses

The research used a survey with multiple-choice questions divided into two parts. The first part covered respondents' age, socioeconomic status and education, while the second part focused on knowledge and attitudes toward endocrine disruptors, including true/false statements based on a previous study (23). The survey gathered information on respondents' awareness of endocrine disruptors, examples like bisphenol A and phthalates, exposure sources and preferred information sources.

The survey related to attitudes and knowledge of mothers in Serbia about endocrine disruptors consisted of three parts. The first part aimed to collect sociodemographic data of the participants, the second part related to knowledge and attitudes about endocrine disruptors, and the third part focused on the sources of information about endocrine disruptors. The total number of respondents was 190. We conducted a sensitivity power analysis, and it suggested that the sample of 190 will be sufficient to observe correlations of 0.2 and higher, with 80% power and a significance threshold of 0.05.

The raw data, i.e., answers to questions testing the level of knowledge concerning endocrine disrupters (EDs), were recorded in order to calculate the number of correct answers. The ‘I don't know’ and ‘I cannot estimate’ options, where present, were always coded as incorrect answers. This decision was made because a choice of either these two alternatives represents a clear indication of a lack of knowledge needed to provide an answer to a particular question. Thus, we report only on the number of correct answers and use this as an indicator of the level of knowledge in participants. There were no missing data, as the respondents were not allowed to skip any questions. We calculated five separate knowledge scores for each question and a total knowledge score as a sum. The data were analyzed using descriptive statistics. Accordingly, we report on distributions: frequencies of chosen alternatives and percentages of correct answers. We also report means and standard deviations, where applicable. We used Chronbach’s alpha to check for scale reliability and Pearson correlation to test the relations between interval variables. To test for group differences, we employed one-way ANOVA. In addition, we conducted the reliability analyses of the ED knowledge scale containing the five aforementioned questions.

## Results

Overall, the majority of the participants (66.8%) had heard the term ‘ED’ before (for distribution, consult Table 2), while one-third (33.2%) of the sample had never encountered it. Among participants familiar with the term (*n* = 127), 42.41% knew precisely what the term referred to, 60.63% reported having ‘some idea about what the term refers to’, while the remainder of the sample (14.96%) had only encountered the term but did not know what it pertained to.

**Table 2:** Frequency of familiarity with the term 'endocrine disrupter' (ED).

	Counts	% of total
No	63	33.2%
Yes, but I don’t know what it refers to	19	10.0%
Yes, and I have some idea of what it relates to	77	40.5%
Yes, and I know precisely what it refers to	31	16.3%

After they indicated whether they have heard of endocrine disruptors, the respondents were provided a short definition of the concept – they were told those were the chemicals that interfere with the normal functioning of glands. Following this, participants completed the rest of the questionnaire.

When asked to assess the risks of EDs, 80.5% of women estimated the health risks of exposure to EDs as high, 2.6% estimated it as low and 16.8% reported that they could not make estimation (Table 3).

**Table 3:** Frequency of familiarity with ED risk assessment.

	Counts	% of total
High	153	80.5%
Cannot assess	32	16.8%
Low	5	2.6%

In addition, our data show that almost all women (93.2%) think that they should get additional information about EDs before pregnancy. In comparison, only 3.2% feel that all information about EDs is readily available and that no special efforts should be allocated to searching for information regarding this topic (Table 4).

**Table 4:** Timing of ED information.

	Counts	% of total
During pregnancy	7	3.7%
When planning pregnancy	177	93.2%
There is no need for additional information; everything is widely available	6	3.2%

Similarly, 85.3% think it is very important that their partner gets informed on EDs as well, while only 5.8% do not care and 9% believe that their partner’s knowledge about the topic is of little or no relevance (Table 5).

**Table 5:** Importance of partner being ED-informed.

	Counts	% of total
Very important	162	85.3%
Neither nor	11	5.8%
Of little importance	14	7.4%
Of negligible importance	3	1.6%

Further, we asked participants how much EDs should be a topic of talk directed at the general public. Two-thirds of the participants (65.3%) think that this topic should be covered a lot more, 28.9% believe that EDs should be talked about more, while 2.1% feel that there is no need to cover EDs more than is already the case (either because the topic is covered enough: 1.6% or ‘there are more significant problems to worry about’ 0.5%). Only 3.7% had no opinion on the subject (Table 6).

**Table 6:** Importance of the public being ED-informed.

	Counts	% of total
It should be talked about way more	124	65.3%
Should be talked about more	55	28.9%
Cannot assess	7	3.7%
It has been talked about already	3	1.6%
Should not be talked about; we have more significant problems	1	0.5%

When asked to assess their previous knowledge about EDs, almost half of the participants reported having no knowledge about EDs before partaking in the present study (47.4%). Another 41.2% of participants informed themselves by individual research on the topic, 7.9% gained information through formal or informal education and courses, and 11.1% were healthcare professionals. Finally, one (0.53%) participant reported having gained knowledge about endocrine disruptors ‘along the way’, and one said that their ability on the topic could be reduced to ‘having information, being aware that the endocrine disruptors exist’. Please note that these percentages do not add up to 100% because participants were allowed to choose more than one option.

Regarding knowledge about EDs, participants were asked how EDs can enter the organism (for the distribution of correct answers, consult Table 7) and the types of products that potentially contain EDs (Table 8). These substances have effects of EDs (Table 9) and implications that best describe EDs (Table 10). Finally, we presented them with a series of statements about EDs (their effects and recommendations for parents and pregnant women), and their task was to select the correct ones.

**Table 7:** Distribution of correct answers to questions on how EDs enter organisms.

Number of correct answers	Number of participants	% of total
0	30	15.8%
1	4	2.1%
2	9	4.7%
3	26	13.7%
4	30	15.8%
5	26	13.7%
6	65	34.2%

**Table 8:** Distribution of correct answers to questions on products that may contain EDs.

Number of correct answers	Number of participants	% of total
0	14	7.4%
2	1	0.5%
3	2	1.1%
4	5	2.6%
5	7	3.7%
6	10	5.3%
7	8	4.2%
8	5	2.6%
9	13	6.8%
10	14	7.4%
11	10	5.3%
12	11	5.8%
13	9	4.7%
14	9	4.7%
15	14	7.4%
16	9	4.7%
17	10	5.3%
18	11	5.8%
19	6	3.2%
20	6	3.2%
21	7	3.7%
22	4	2.1%
23	5	2.6%

**Table 9:** Distribution of correct answers to questions on substances known to have ED effects.

Number of correct answers	Number of participants	% of total
0	39	20.5%
1	10	5.3%
2	16	8.4%
3	26	13.7%
4	26	13.7%
5	30	15.8%
6	14	7.4%
7	8	4.2%
8	6	3.2%
9	4	2.1%
10	11	5.8%

**Table 10:** Distribution of correct answers on questions on implications that best describe EDs.

Number of correct answers	Number of participants	% of total
0	1	0.5%
4	1	0.5%
5	2	1.1%
6	5	2.6%
7	8	4.2%
8	9	4.7%
9	14	7.4%
10	16	8.4%
11	25	13.2%
12	25	13.2%
13	24	12.6%
14	28	14.7%
15	20	10.5%
16	10	5.3%
17	2	1.1%

Our data show that 34.2% of participants correctly selected all six potential sources of EDs (how EDs can enter the organism), 13.7% missed one, 15.8% missed two sources, and one-third of participants scored three or lower on this question (15.8% had zero correct answers). The mean number of correct answers was 3.89 (SD = 2.135). Regarding types of products that possibly contain EDs, participants had zero (7.4%) to 23 (all; 2.6%) correct answers (*M* = 11.93, SD = 6.075). Among the alternatives offered, the most commonly selected options were deodorants and household cleaning products – 86.3 and 82.6% of the sample correctly selected these options, respectively. The results also showed that 70% of participants or more selected shower gels (74.2%), perfumes (71.6%), makeup (70.0%), air fresheners (72.6%) and plastic packaging (73.2%). Over half of the participants knew that bottled water (51.1%), facial creams (68.4%), baby creams (57.9%), diapers (50.0%), drugs (59.5%), air (52.1%), toys (52.6%) and scented candles (56.8%) potentially contain EDs. However, most of the participants did not know that different kinds of packaging (aluminum: 32.6 correct; glass: 4.2% right; cardboard: 20.5% accurate and printed: 35.3% correct), jewelry (24.2% correct), furniture (22.6% correct), fresh fruit and vegetables (46.3% correct) and tap water (28.4% accurate) also possibly contain EDs.

Regarding substances known to have effects of EDs, the mean number of correct answers was 3.73 (SD = 2.854). Among the ten alternatives offered, only 5.8% of participants correctly selected all, while 20.5% had zero correct answers. Over 50% of participants knew that parabens (58.9%), pesticides (57.4%) and toxic metals (53.7%) had effects on EDs. Over one-third of participants knew that bisphenol A (45.3%) and phthalates (38.4%) act as EDs. Finally, the options that were selected the least amount of times were alkylphenol (18.4%) and flame retardants (16.3%), followed by nitrates (27.9%), PCBs (25.3%) and phytoestrogens (31.1%).

Our data show that participants don’t have adequate knowledge of the categories of substances that best represent EDs. The mean number of correct answers was 1.94 (SD = 0.955). Only 2.6% of participants correctly selected all suitable alternatives, while four times as many (9.5%) had zero correct answers. Almost half of the participants (45.3%) had two out of four correct answers, and 17.4% gave only one right answer.

The knowledge regarding risks and recommendations regarding EDs varied among participants (*M* = 11.74, SD = 2.871). 1.1% (two participants) gave all correct answers, while 0.5% (one participant) gave no correct answers. Most participants (64.2%) gave correct answers between 11 and 15 (out of 17). In line with this is the finding that only one-third of participants (39.5%) felt that they knew how to reduce exposure to EDs. In addition, only 39.5% reported understanding how to minimize exposure to products containing endocrine disruptors.

Regarding sources of information, i.e., whom women would like to hear more about EDs from, most of the participants think that should be a general practitioner (81.05%), followed by a gynecologist (71.05%), an endocrinologist (66.84%) and a pharmacist (64.74%). A small number of participants (5.26%) added media to the open-ended option. Finally, only 3.68% of women feel that no additional education on the topic of EDs is necessary.

Cronbach’s alpha of the scale was 0.715. In addition, we conducted an exploratory factor analysis with the knowledge scale items and obtained a one-factor solution, with all items loading higher than 0.50 (varying between 0.52 and 0.84), explaining 45.5% of the total variance. This allowed us to compute a total ED knowledge score. In the next step, we correlated that score with age. Pearson’s r was non-significant (*r*(199) = 0.053; *P* = 0.468). We have also conducted a one-way ANOVA to test whether there were differences between groups with different educational levels by total ED knowledge and observed the differences in the expected direction – knowledge was the highest among participants with a Ph.D. (*M* = 38.0, SD = 3.05). It linearly decreased, so it was the lowest among participants who only finished high school (*M* = 27.3, SD = 2.11), (*F* (4, 32) = 3.03; *P* = 032). We have also observed the expected knowledge differences by the familiarity of the term ED, *F* (3, 64) = 35.4: *P* < 0.001, so that those reporting they were more familiar with the term were more knowledgeable, and by ED risk assessment, *F* (3,10) = 21.4: *P* < 001, so that those who reported they could not assess the risk were the least knowledgeable.

## Discussion

Previous research has examined the knowledge of the general population (24) and perinatal health professionals (4), as well as public perceptions of risks related to endocrine disruptors in drinking water (25). Only a few studies have explored the awareness of endocrine disruption among mothers and pregnant women (22, 26, 27, 28, 29, 30, 31, 32). The current study represents the first survey assessing the knowledge, attitudes, and ways of informing mothers about endocrine disruptors conducted in Serbia. The findings are significant due to rising pollution and increasing infertility rates in the region (33, 34, 35, 36). Furthermore, disparities in education and income, coupled with the increasing prevalence of infertility in Serbia, enhance the importance of these findings (37, 38).

### Sociodemographic data of the participants

Our study showed that the average age of the respondents was 38.34 years. Also, most participants live in an urban area and have finished undergraduate university studies or master’s studies. Compared to respondents from other studies (26, 28), our study showed an increased average age. This is particularly important since age represents a significant risk factor for infertility (39). The postponement of fertility, as evident in the alterations in the age distribution of childbirth, especially for first-time parents, mirrors various shifts in employment, educational trends and lifestyle, such as living in urban areas (37). More extended education may have been an additional reason for the age of the respondents observed in our study, as it has been suggested that changes in education patterns contribute to the rise in the average age at which women have children (37). On the other side, according to the report of the European Union, the mean age for women to have their first child was 29.5 years in Serbia in 2021 (https://doi.org/10.2908/TPS00017). The exact age was the mean age for women to have their first child in the European Union (comprising 27 countries as of 2020) (39). This may be because our study included mothers with older children, not just pregnant women or mothers of newborns. In addition, we did not account for whether it was their first pregnancy, and the sample size was limited. However, age and education are crucial factors in assessing risk perception of EDCs, as both were found to increase EDC risk awareness (29).

### The knowledge and attitudes about endocrine disruptors

EDCs have garnered significant attention in recent scientific literature due to their concerning implications on female fertility and children’s health. Endocrine disruptors have the capability to disrupt the regular functioning of hormones, metabolism and biosynthesis, leading to alterations in the natural hormonal balance. For instance, studies have shown that pesticide exposure may reduce fertility and cause abnormal births. In addition, exposure to pesticides might raise the risk of reproductive problems such as irregular menstruation, reduced fertility, preterm birth, spontaneous abortion, congenital abnormalities and low birth weight, among others (40). Investigations into bisphenol A, which is prevalent in plastics and epoxy resins, have highlighted its potential to interfere with ovarian function and compromise female fertility by binding to the ER and activating or inhibiting estrogenic function (41). Phthalates, widely used in plastics and personal care products, have been associated with gestational diabetes, preterm birth, obesity and decreased body mass index in children (42, 43). Benzophenone derivatives, which are used in personal care products, cosmetics, sunglasses, plastic packaging and sunscreens as UV protectors, can affect the duration of pregnancy and thus fetal growth during early and late stages of pregnancy (44). The presence of PCBs in the environment has been linked to disruptions in menstrual regularity and diminished fecundity (45). Flame retardants, commonly present in household items, have shown adverse effects on ovarian function and hormone levels, potentially impacting fertility outcomes (46). Some studies have suggested that exposure to alkylphenols may be associated with adverse effects on female reproductive health, including disruptions in menstrual cycles and alterations in hormone levels. Oral contraceptives, often overlooked as an environmental risk factor, may potentially contribute to increased autism spectrum disorder (ASD) prevalence through their disruption of endogenous endocrine function and subsequent neurodevelopmental effects, suggesting a need for further investigation into their epigenetic and transgenerational impacts (11, 12).

Considering all the aforementioned facts, our survey aimed to assess the measure of familiarity with the term ‘endocrine disruptors’, the depth of understanding, the sources of information, and the perceived significance in that field. Interestingly, most participants (66.8%) had heard of ‘ED’ before, but only 42.41% knew precisely what the term refers to. Regarding their previous knowledge about EDs, most respondents did not know about EDs before participating in the present study (47.4%), or they informed themselves by individual research (41.2%). Our data show that the respondents do not have adequate knowledge of potential sources of endocrine disruptors (34.2% of respondents selected all potential sources), alternatives (5.8% of respondents correctly selected all), and categories of substances (only 2.6% of respondents correctly selected all). These results were in line with previously conducted studies in France (28) and Canada (27), supporting the need for providing educational materials for pregnant women and perinatal health professionals regarding endocrine disruptor exposure.

In addition, our study showed that 80.5% of respondents estimated the health risks of exposure to endocrine disruptors as high. The vast majority of respondents (93.2%) think they should get additional information about endocrine disruptors before pregnancy. Also, 85.3% feel that it is essential that their partner gets informed on this information.

This underscores the necessity for educational initiatives in this domain and aligns with the recommendation that healthcare professionals should take into account the knowledge and perceptions of pregnant women with caution in order to avoid raising their anxiety while providing advice regarding EDCs and environmental exposure (29).

### True/false statements

During pregnancy, exposure to tobacco smoke is detrimental to fetal growth and development, resulting in adverse birth outcomes, including preterm birth, low birth weight and increased perinatal and infant mortality rates (47). Furthermore, it is important to note that indoor air quality can often be more compromised than outdoor air in this context (47). EDCs in numerous consumer products hold particular significance, as specific processes can influence the extent of exposure (13, 48). For instance, heating plastic food-contact materials may expedite the release of these compounds (49). Therefore, opting for alternatives whenever feasible, such as using glass baby bottles instead of plastic ones or selecting less skin-permeable personal hygiene products, is advisable (50, 51).

Moreover, making dietary choices, such as consuming organic foods, can reduce exposure to endocrine disruptors (52). Additional precautions involve limiting fatty fish intake and peeling fruits and vegetables to reduce pesticide residues (9). Moreover, exposure to endocrine disruptors during the prenatal period affects the developing fetus at a stage when organogenesis is yet to be fully completed (20, 53). Endocrine disruptors have the potential to alter early developmental processes and increase susceptibility to disease/dysfunction later in life (54).

Considering the significance of the previously mentioned characteristics of EDCs, which may not always be readily inferred, an assessment of the respondents' awareness in this domain was conducted. The understanding of risks and recommendations concerning endocrine disruptors differed among the participants in the current survey. However, most respondents gave between 11 and 15 (out of 17) correct answers, which could be attributed to their level of education (58.9% have finished undergraduate university studies or master’s studies). Women with higher levels of education are inclined to have improved access to environmental health information, possess enhanced capabilities to comprehend and analyze this information, and often have more resources at their disposal to underpin their lifestyle choices (55, 56). Given that merely a third of the participants expressed confidence in their ability to mitigate exposure to endocrine disruptors and only 39.5% have reported knowledge about reducing exposure to products containing such disruptors it underscores the need for targeted educational initiatives addressing exposure, associated risks and recommended preventive measures in future efforts.

### The sources of information about endocrine disruptors

It is known and has already been stated that knowing the type and source of endocrine disruptors in our environment is an essential step that can lead to a reduction in exposure and, consequently, the potentially harmful effects that may arise. It is imperative whether the sources we look for information are adequate, especially nowadays when the general population is exposed to different sources of information, reliable and unreliable. Mothers must know about endocrine disruptors, especially concerning the health and well-being of their children. These chemicals may impact the developing fetus and children during their early years or puberty (54, 57). In this regard, the significance of minimizing exposure during these critical stages could be accomplished through the education of mothers, considering their pivotal role within the family unit. Through the questions from the survey questionnaire, which were related to the sources of information, we wanted to look at the perception of mothers about the importance of this topic for them.

Most mothers (93.2%) believe they should be informed about endocrine disruptors before pregnancy, while only 3.2% feel current information is sufficient. This highlights concerns about exposure and its impact on children’s health, emphasizing the need for proper education to reduce exposure. When asked who should provide this information, most mothers pointed to general practitioners, gynecologists, endocrinologists and pharmacists. A few mentioned the media, and a small percentage felt such education was unnecessary. The fact that mothers trust health professionals the most regarding this topic and expect answers from them also raises the question of the health professionals being informed in this area. A recent study conducted in France (year 2015), in which a total of 189 healthcare professionals participated, showed that only 17% of healthcare professionals felt ready to answer questions about phthalates in pregnant women (58). Another study conducted in 2018 aimed to assess the knowledge of perinatal health professionals about endocrine disruptors. Out of 1650 respondents who completed the questionnaire, only 181 believed they had adequate knowledge about endocrine disruptors. Moreover, the majority of healthcare professionals stated that they do not provide information to women on this topic during pregnancy (4). A review paper that considered the role of physicians in disseminating information about environmental pollutants as factors affecting human health (except for tobacco smoke) unequivocally showed that physicians do not provide enough of this information but are aware of the risks of exposure to pollutants (59).

Regarding Serbia, no studies would indicate health professionals' knowledge about the exposure and harmful effects of endocrine disruptors. Due to the complexity of the topic and the need to convey knowledge to mothers using adequate terminology without causing fear, we believe that doctors in our country do not pay enough attention to this area.

When it comes to the familiarity of the general population with the sources and effects of endocrine disruptors, it has also been shown that awareness is low. As a solution, it was proposed to increase public awareness through the media, providing education to health professionals and health education in schools (10).

To the question from the questionnaire, ‘Is it important for your partner to be informed about endocrine disruptors?’ 85.3% of mothers think it is important for their partner to be informed about it. Informing the partner can be significant because, in this way, the pregnant woman feels safer about the joint effort to reduce exposure to harmful chemicals.

Being a survey-based study, there are, of course, certain limitations to this study. The sample size could be more significant to better represent geo-economical differences among the mothers in Serbia, reflecting the knowledge differences. Furthermore, it is hard to interpret the results in light of the studies conducted in other countries due to differences in healthcare systems, education systems and regulations. Also, it should be noted that the population surveyed is predominately limited to higher-educated mothers living in urban areas of Serbia, thus not sufficiently representing the general population.

## Conclusions

Considering the differences in levels of knowledge about endocrine disruptors among the general population, including mothers and pregnant women worldwide, this survey contributes to the overall identification of knowledge gaps. Although not necessarily reflecting the general population since the survey was limited to mainly those with higher education and living in urban areas, our results still indicate that mothers in Serbia do not have adequate knowledge about endocrine disruptors (potential sources, categories of substances, alternatives) or confidence in their ability to mitigate exposure to endocrine disruptors. On the other side, the estimation of the health risks of exposure to endocrine disruptors was perceived as high, and the mothers thought that they should get additional information about endocrine disruptors before pregnancy. The most significant percentage of respondents believes that their partner should be better informed on this information. The aforementioned results indicate a greater need for mothers' education in Serbia to dispel any concerns about endocrine disruptors. Our results also suggest that education provided by health professionals (mainly general practitioners, then gynecologists, endocrinologists and pharmacists) would be the most appropriate, considering the high degree of trust shown by the respondents. Hence, further steps would include carefully designing evidence-based educational programs for healthcare professionals in the field. With successful validation, the educational paradigm has the potential for adaptability and scalability across many target groups, informing them about the risks associated with chemicals in food, cosmetics and lifestyle choices and providing practical strategies to minimize exposure. Moreover, the educational program would have the potential to be seamlessly included in prenatal care, serving as a complementary component to routine medical check-ups, nutrition counseling and birthing lessons.

## Declaration of interest

The authors declare that there is no conflict of interest that could be perceived as prejudicing the impartiality of the research reported.

## Funding

The work is financed by the project evaluating the effectiveness of a perinatal environmental health educational intervention in reducing maternal and infant exposure to EDCs (Mie-Rex) funded by the NYU CEHRT Pilot projects, NIEHShttps://doi.org/10.13039/100000066, USA and the project Chemical Exposure Reduction: Empowering Women through Education and Choice (CH-EMPOWER, project no 14907) funded by The Science Fund of the Republic of Serbiahttps://doi.org/10.13039/501100016047.

## Author contribution statement

KŽ contributed to conceptualization, methodology, formal analysis, investigation and writing, reviewing and editing the original draft, as well as visualization. ĐM contributed to formal analysis, investigation and writing the original draft LM was involved in data curation, investigation and writing the original draft. VB participated in formal analysis, investigation and writing the original draft JŽ contributed to data curation, investigation and writing the original draft. DDC, ZB and BA contributed to methodology, writing, reviewing and editing. ZV was responsible for data curation, investigation and writing the original draft. SI contributed to data curation, formal analysis and investigation. IŽ helped in formal analysis, investigation and in writing the original draft. ABDJ was responsible for conceptualization, methodology writing, reviewing, editing, supervision, resources, validation, funding acquisition, project administration.

## Ethical approval

Ethical permission was obtained from the Ethics Committee for Biomedical Research of the Faculty of Pharmacy, University of Belgrade, Serbia. (no 220/2).
